# The Impact of Climatic Risk Factors on the Prevalence, Distribution, and Severity of Acute and Chronic Trachoma

**DOI:** 10.1371/journal.pntd.0002513

**Published:** 2013-11-07

**Authors:** Anita Ramesh, Sari Kovats, Dominic Haslam, Elena Schmidt, Clare E. Gilbert

**Affiliations:** 1 International Centre of Eye Health (ICEH), Clinical Research Department, Faculty of Infectious and Tropical Diseases, London School of Hygiene and Tropical Medicine (LSHTM), London, United Kingdom; 2 Department of Social and Environmental Health Research, Faculty of Public Health and Policy, London School of Hygiene and Tropical Medicine (LSHTM), London, United Kingdom; 3 Sightsavers International, Haywards Heath, United Kingdom; Centers for Disease Control and Prevention, United States of America

## Abstract

**Background and Objectives:**

Trachoma is the most common cause of infectious blindness. Hot, dry climates, dust and water scarcity are thought to be associated with the distribution of trachoma but the evidence is unclear. The aim of this study was to evaluate the epidemiological evidence regarding the extent to which climatic factors explain the current prevalence, distribution, and severity of acute and chronic trachoma. Understanding the present relationship between climate and trachoma could help inform current and future disease elimination.

**Methods:**

A systematic review of peer-reviewed literature was conducted to identify observational studies which quantified an association between climate factors and acute or chronic trachoma and which met the inclusion and exclusion criteria. Studies that assessed the association between climate types and trachoma prevalence were also reviewed.

**Results:**

Only eight of the 1751 papers retrieved met the inclusion criteria, all undertaken in Africa. Several papers reported an association between trachoma prevalence and altitude in highly endemic areas, providing some evidence of a role for temperature in the transmission of acute disease. A robust mapping study found strong evidence of an association between low rainfall and active trachoma. There is also consistent but weak evidence that the prevalence of trachoma is higher in savannah-type ecological zones. There were no studies on the effect of climate in low endemic areas, nor on the effect of dust on trachoma.

**Conclusion:**

Current evidence on the potential role of climate on trachoma distribution is limited, despite a wealth of anecdotal evidence. Temperature and rainfall appear to play a role in the transmission of acute trachoma, possibly mediated through reduced activity of flies at lower temperatures. Further research is needed on climate and other environmental and behavioural factors, particularly in arid and savannah areas. Many studies did not adequately control for socioeconomic or environmental confounders.

## Author Summary

Trachoma – the leading cause of infectious blindness – is spread through contact with infected persons by hands and towels, and by ‘eye-seeking flies.’ Trachoma prevalence is high in areas characterised by poverty, inadequate water supply, and poor sanitation. Trachoma is controlled by the SAFE strategy: S = surgery to the upper eyelids; A = antibiotics for active infection; F = facial cleanliness; and E = environmental improvement. In this study we reviewed the scientific literature to assess the extent to which climatic factors (e.g., rainfall, heat, dust, altitude) influence trachoma distribution. A systematic review of the literature found eight papers that measured an association between a climatic factor and trachoma in children or adults. Several studies reported that trachoma is less common at higher altitudes, indicating that temperature may play a role in trachoma transmission. Some studies also reported that trachoma is higher in areas with low rainfall, which is consistent with anecdotal evidence that trachoma is associated with dry environments.

## Introduction

The neglected tropical disease (NTD) trachoma, caused by *Chlamydia trachomatis*, is the world's leading cause of infectious blindness [1] and an important cause of chronic discomfort in 57 endemic countries, mainly in Africa [2]. Over 40 million people are infected with *C. trachomatis*, 8 million of whom have painful, in-turned eyelashes (trichiasis) [3]. It is estimated that 1.2 billion people live in trachoma-endemic areas [4]. The current distribution of trachoma aligns with low and middle-income countries, within which poorer individuals and communities are at highest risk [2], [5].

The World Health Organization (WHO) classifies trachoma into active and chronic stages. The signs of active trachoma, according to the simplified grading system [6], are trachomatous follicles (TF) and trachomatous inflammation (TI). The chronic, potentially blinding stages of trachoma are characterised by visible scarring of the under surface of the upper eyelid (TS), in-turned eyelashes, trachomatous trichiasis (TT), and corneal opacity (CO). The incidence of chronic trachoma increases with age. Active infection principally occurs in children, where it is self limiting, whereas the blinding stages of trachoma are not seen until later in life. Although repeated episodes of infection during childhood are thought to lead to the scarring stages of trachoma, the natural history and pathophysiology are not fully understood. Both clinical presentation and transmission of trachoma are complex and multi-faceted. The investigation and monitoring of active trachoma are complicated as evidence of infection with *Chlamydia* does not correlate well with clinical signs of the disease (i.e. TF and TI) [7].

Trachoma is transmitted via contact with infected eye and nasal secretions by hands, fomites, and eye-seeking flies [8]. Individual factors such as rural residence, overcrowded living conditions, and keeping cattle close to the home are also associated with trachoma [5], [9]–[11]. The strongest environmental risk relates to poor hygiene, often reflecting poor access to water and lack of sanitation, which promotes transmission by providing more effective breeding sites for eye-seeking flies [12]–[17]. The strategy for trachoma control is the SAFE strategy: S = surgery to correct the upper eyelid deformity [18]; A = antibiotics for active infection via mass drug administration (MDA) [19]; F = facial cleanliness [20], and E = environmental improvement [21]. The ‘F’ and ‘E’ elements aim to reduce *C. trachomatis* transmission by improving hygiene behaviour and reducing environmental factors which promote eye-seeking flies.

There has been growing interest in the direct effects of climate on disease, reflecting concerns about climate change as well as improvements in the use of climate information for disease control [22]. Epidemiological studies of weather (daily temperature or rainfall) use time series methods to detect acute (short-term) effects on health outcomes. Long term exposures (i.e. climate) have also been studied in cross-sectional studies [23]. Climate factors vary in time and space and it is important that the study design includes appropriate adjustment for social or environmental factors (confounders). Associations between meteorological variables and health outcomes are likely to depend on local contexts, and have also been shown to change over time [22]. Climate is the average weather conditions observed over a long time period (decades). Globally, the world can be divided into five main climate types (with multiple sub-types) based on annual average and monthly temperature and precipitation values. The best known classification scheme is the Koppen-Geigen system which describes the following climate types: tropical; dry (arid and semiarid); mild temperate; continental and polar [24].

Climatic factors may influence the distribution and prevalence of trachoma indirectly through poor access to water, which limits hygiene behaviour, or low rainfall, which may influence the distribution, abundance or seasonal activity of *Musca sorbens*, the principal eye-seeking fly implicated in trachoma transmission. At broad geographic levels, climate also influences agricultural productivity and livelihoods in resource-poor settings; communities at risk of trachoma often depend on livestock and subsistence farming. Direct influences on the eye may include humidity, dust and aridity.

The purpose of this study was to examine the association between climatic factors and the distribution, frequency and severity of trachoma. A systematic literature review was conducted for evidence of climatic effects on active trachoma, to identify factors associated with transmission, as well as cicatricial/blinding trachoma, to identify factors associated with scarring.

## Methods

Searches were undertaken separately for active and chronic trachoma (hereafter termed trachoma outcomes) (See search terms in Supplementary Material Table S1). The following electronic databases were searched: CAB Abstracts; Embase; Global Health; Medline; Web of Science. Websites of international agencies were also searched: the World Health Organization (WHO), WHO Special Programme for Research and Training in Tropical Diseases (TDR); Intergovernmental Panel on Climate Change (IPCC); United Nations Children's Fund (UNICEF); UN-Habitat; The Carter Center; the International Trachoma Initiative (ITI); Sightsavers; Helen Keller International; Fred Hollows Foundation; Christian Blind Mission.

Studies were only included if they quantified an association between a climate factor (temperature, rainfall, altitude, etc) and a trachoma outcome. Papers were not excluded based on geographic location of study, age of participants, or language of journal publication. Peer reviewed journal articles and reports from leading international agencies (e.g., WHO) published between 1 January 1950 and 1 April 2012 were included.

Data extraction and analysis were conducted by two readers. Results were screened and reviewed in three stages: i) title; ii) title and abstract; and iii) title, abstract, and manuscript. References for which title and abstract were available and which seemed to meet the inclusion criteria were reviewed by two reviewers for eligibility, quality and data extraction. The quality of each study included was assessed using the parameters described in the STROBE checklist for cross-sectional studies [25]. The quality of observational studies was assessed using the following criteria: sampling of study population to estimate trachoma prevalence, study design and control of confounding, reporting of negative results, and the measurement of climate exposures.

It was expected that heterogeneity in the study designs and exposure measurements would preclude meta-analyses. We also reviewed the evidence for climate type and seasonality on trachoma outcomes.

## Results

**Figure 1:**
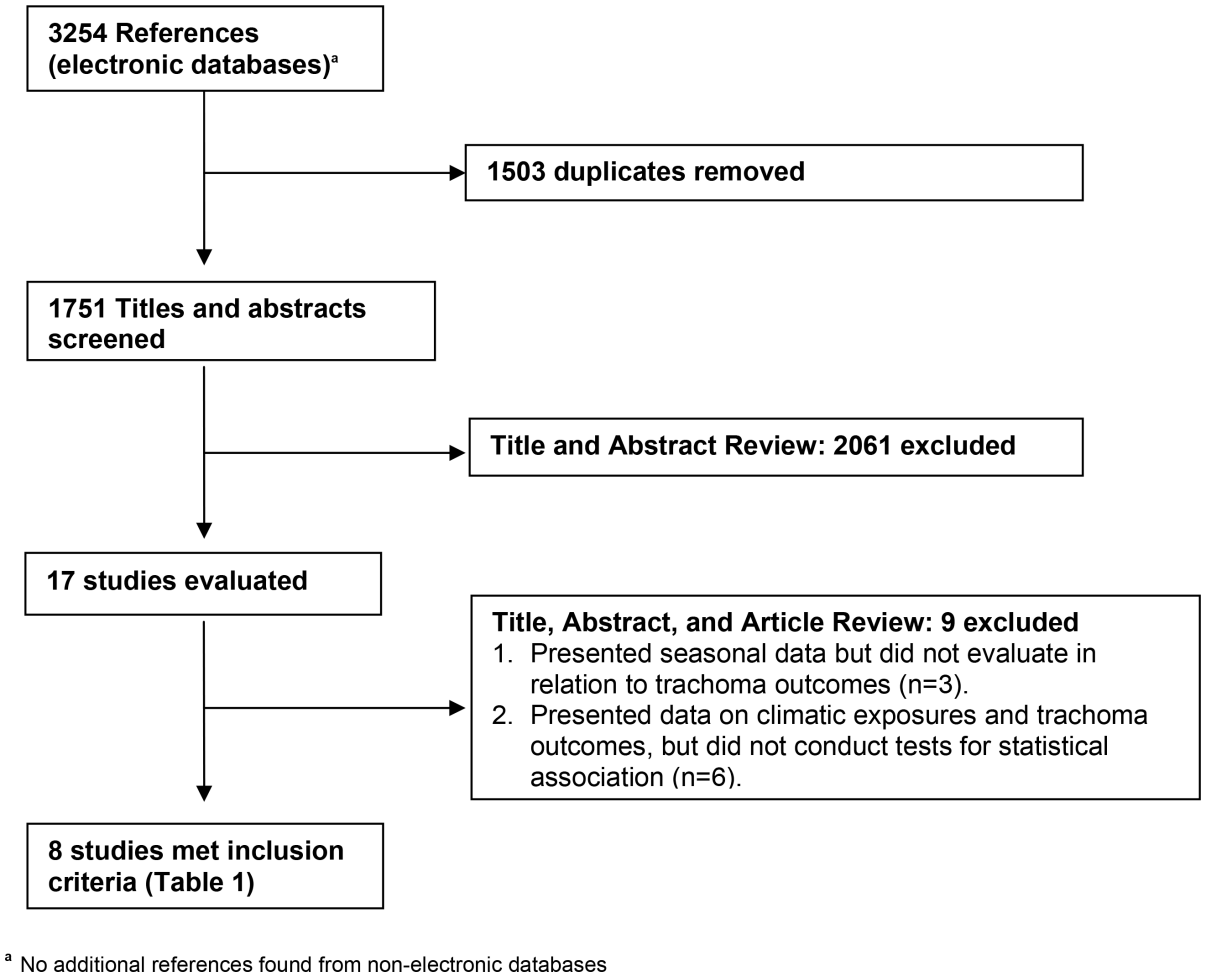
Study screening and selection process.

Only eight of the 1751 papers retrieved met the inclusion criteria (Figure 1, Table 1) [17], [26]–[32]. All studies were cross-sectional and all used the WHO simplified grading system for trachoma, which requires use of a ×2.5 magnifying loupe while examining the under surface of the upper eyelid to give more detail of pathological changes. However, use of magnification was inconsistent or not reported. All eight studies had been published since the year 2000, although field work in one study had been undertaken earlier [28]. All were undertaken in African countries, principally across the Sahel belt. Three of the eight studies were conducted in West Africa: Mali, two [26], [28]; Burkina Faso, one [29], and five in East Africa: Ethiopia, three [30]–[32]; Tanzania, one [17] and South Sudan, one [27].

**Table 1:** Evidence of the effects of climate exposures on trachoma outcomes.

Country, Authors and year, study quality	Climate exposure, measure	Trachoma outcome, population	Methods	Results (Prevalence/Odds Ratio, 95% Confidence Interval (CI)) *Where noted CI = Credibility Interval* a
Mali, Schemann et al., 2007: Study quality: moderate	Altitude, low (<260 m), medium (260–350 m), and high (≥350 m); mean daily temperature; latitude, longitude, annual rainfall (eight isohyets classes from 0–1200 mmn)	Active trachoma (TF/TI) in children <10 years; chronic trachoma (TS/TT/CO) in adult women >14 years	Cross-sectional study using clustered survey data. Multivariate analysis included longitude and latitude but adjusted for altitude, mean daily temperature, annual rainfall, and relative humidity. 2.5× loupe use not reported.	**Prevalence: TF/TI** <10 years: 34.9% (32.3, 37.6); **TS:** 23.7% (21.3, 26.1); **TT:** 2.5% (2.1, 2.9); **CO:** 1.0% (0.8, 1.3). **Results for TF/TI:** *Multivariate analysis:* Latitude an independent explanatory factor for TF/TI for latitudes between 13 and 15°N [(0.62 (0.54—0.73) p<0.001], and 0.58 for latitude <13°N; both compared to latitudes >15°N. Mean temp >31°C [1.17 (1.02, 1.34) p>0.026]. **Results for TS/TTCO:** *Multivariate analysis:* Latitude an independent explanatory factor for TS for latitudes between 13 and 15°N [1.93 (1.64–2.28); p<0.001], and for latitudes <13°N [3.72 (3.05—5.27); p<0.001]; both compared to latitudes >15°N. Longitude was not significant and was not kept in the models. Rainfall and relative humidity dropped as co-linear with latitude. Latitude an independent explanatory factor for TT/CO (for latitudes 13–15°N [OR = 1.88 (1.17—3.02); p = 0.009], and for latitude <13°N [4.18 (2.50—6.99); p<0.001]; both compared to latitudes >15°N. Longitude was not significant and was not kept in the models. Rainfall and relative humidity dropped as co-linear with latitude
Southern Sudan Clements et al., 2010. Study quality: moderate	Altitude; interpolated long-term average monthly min/max temperature; average monthly min/max rainfall.	Active trachoma (TF/TI) in children 1–9 years	Spatial mapping. Logistic regression with odds ratios (ORs) and 95% Credibility Intervals, Bayesian methods (interpolation) using the deviance information criterion (DIC) to select the best model. Model 1: fixed effects for age, sex, long-term average annual rainfall, land cover. Model 2: fixed effects as above+geostatistical location-level random effects with a correlation structure defined by an isotropic exponentially decaying autocorrelation function. 2.5× loupe use not reported.	**TF/TI prevalence 48.2% (range in different locations 2.2–77.6%).** *Rainfall: S*ignificant negative correlation between rainfall and active trachoma prevalence. Land cover was a significant explanatory variable in model 1, but not model 2. Rainfall and wetland appear to be protective, while savannah and grassland appear to be associated with risk for TF/TI. *Model 1* Climate Factors [OR (CI)^a^; DIC]: Rainfall: [0.55 (0.49–0.62)]; Land cover/savannah: [1.77 (1.48–2.11)]; Land cover/grass, shrub, cropland: [0.62 (0.50–0.75)]; DIC = 6554.9. *Model 2* Climate Factors [OR (CI);^a^ DIC]: Rainfall: [0.21 (0.08–0.46)]; DIC = 4753.2.
Mali, Hagi et al., 2010. Study quality: moderate	Altitude; rainfall; sunshine fraction; temperature (average monthly max, annual average); sunshine fraction, number of rainy days.	Active trachoma (TF/TI) in children 1–10 years	Secondary cross-sectional analysis of national trachoma survey. Bayesian hierarchical logistic models: iterative generalized least square model(IGLS) with 95% confidence intervals or Bayesian hierarchical model (BHM) with 95% credibility intervals. 2.5× loupe use not reported.	**TF/TI prevalence:** 35.0% (34.2%–35.8%).^a^ *Average monthly temperature:* Relative to areas of <34.6°C, areas of 34.6–38.7°C had lower TF/TI prevalence: IGLS [0.56 (0.37–0.87)] and BHM [0.51 (0.29–0.0.90)^a^]. Areas of >38.7°C had higher prevalence: IGLS [1.02 (0.61–1.70)] and BHM [1.03 (0.51–2.05)^a^]. *Annual average temperature:* Relative to annual temperature of <27.3°C, areas of 27.3–28.1°C: IGLS [0.54 (0.40–0.74] and BHM [0.49 (0.32–0.74)^a^]. Areas of >28.1°C: IGLS [0.63 (0.45–0.89)] and BHM [0.57 (0.36–0.90)^a^] *Average monthly sunshine fraction (SF):* Relative to <62.8%, areas with SF 62.8–69.9%: IGLS [0.66 (0.47–0.92)] and BHM [0.61 (0.41–0.91)^a^] while a SF of >69.9%: IGLS [0.55 (0.38–0.79)] and BHM [0.50 (0.32–0.79)^a^]. *No of rainy days/month:* Relative to no rainy days, areas with ≥1 rainy day per month: IGLS [0.63 (0.41–0.97)] and BHM [0.57 (0.31–1.07)^a^].
Burkina Faso, Koukounari et al., 2011. Study quality: moderate	Altitude, precipitation, min temperature, max temperature, average air pressure, air pressure	Active trachoma (TF/TI) in children aged 1–9 years	Binomial logistic regression models and Markov-Chain Monte Carlo (MCMC); variables included by backward stepwise elimination. 2.5× loupe use not reported.	**TF/TI prevalence** 13.30% (12.14%, 14.45%).^a^ **Results:** Simple hierarchical logistic regression models – Odds Ratios: Altitude: 1.730 per 50 MSL (1.189–2.433);^a^ Precipitation: 1.690 per 0.5 m (1.336–2.016);^a^ Minimum temperature: 0.570 per 0.5 deg C (0.546–0.591); Maximum temperature: 0.465 per 1 deg C (0.436–0.484); Average temperature: 0.580 per 0.5 deg C (0.564–0.591); Air pressure per 5 mbars: 0.606 (0.602–0.609). Final multivariable hierarchical logistic regression model: factors after backward elimination, Minimum temperature per 0.5 deg C: 0.746 (0.717–0.768); and air pressure: 0.616 per 5 mbars (0.608–0.622).
Ethiopia, Haileselassie and Bayu, 2007. Study quality: low	Altitude: low (<1800 m), medium (1800–2449 m), and high (≥2500 m)	Active trachoma (TF/TI) in children 1–10 years	Stratified cluster sampling via 3 strata: low, medium, and high altitude. 2.5× loupe and torch used (but loupe magnification not specified).	**TF/TI Prevalence:** 52.4% (TF 41.3% and TI 21.4%). **Results**: Altitude: TF/TI were significantly associated with altitude, with both greater in lower than higher altitudes: TF prevalence in low (42.3%) vs. high (17.3%) altitudes; TI prevalence in low (36.5%) vs. high (4.2%) altitudes; X^2^: 267.88; Fisher's exact p<0.001.
Ethiopia. Ngondi et al., 2008. Study quality: low	Altitude: <1500–2000 m;2001–2500 m; >2500 m	Active trachoma (TF/TI) in children 1–9 years; chronic trachoma in (TT) in adults ≥15 years	Hierarchical regression using generalized linear models (GLMs); ordinal and normal logistic (stepwise) regression, with final multivariate model adjusted for age and sex. 2.5× loupe use not reported.	**Prevalence: TF** & TI (children) = 24.9% and 21.9%; **TT** (adults) = 6%. **Results for TF/TI:** Test for trend across categories P_trend_ <0.001 <1500–2000 m OR = 1.8(1.2,2.6). 2001–2500 m: OR = 2.7 [95% CI 1.8, 4.1]; >2500 m: OR = 3.8 [95% CI 2.3, 6.4]. **Result for TT:** Test for trend across categories P_trend_ <0.015 [<1500–2000 m, OR = 1.7 (1.1–2.9) 2001–2500 m: OR = 2.5 [95% CI 1.5,4.1]; >2500 m OR = 1.4 (0.7–2.6); altitude of >2500 m OR = 1.4 [95% CI 0.7, 2.6]
Ethiopia, Alemayehu et al., 2005. Study quality: low	Altitude: 1800–2000 m;2001–2200 m; 2201–2400 m; 2401–2600 m; 2601–2800 m; 2801–3000 m; >3000 m. Latitude and longitude	Active trachoma (TF/TI) in children 1–6 years	Cross sectional analysis using multistage cluster sampling survey data. 2.5× loupe used.	**TF/TI prevalence: 56.5% (54.7%, 58.3%). Altitude:** Children living at <2000 m had highest prevalence of TF/TI (73.4%); children living at higher altitudes had lowest prevalence (5.7%) (X2: 687.74; Fisher's exact p<0.001). **Latitude:** no association.
Tanzania, Baggaley et al., 2006. Study quality: low	Altitude [quartiles]: 822–1337.3 m; 1337.4–1514.8 m; 1514.9–1703.8 m; 1703.9–2268.5 m	Active trachoma (TF/TI) in children 1–9 years	Cross- sectional analysis using national survey data. Logistic regression, adjusted for clustering of cases within households. 2.5× loupe use not reported.	**TF/TI prevalence 13.30% (12.14%, 14.45%). Altitude:** Acute trachoma and altitude were inversely related which remained after adjustment for confounders (adjusted OR for last quartile compared to first quartile 0.56 (0.41, 0.76). Increasing altitude and distance to water were both associated with acute trachoma (age-adjusted P for trend <0.0001 for each)

a 95% credibility intervals.

Four of the eight studies were judged to be of moderate quality [26]–[29] and four of lower quality [17], [30]–[32]. The four moderate quality studies provided the best and most comprehensive measurement of climatic exposures, used a more detailed methodology and complex analyses. Three of these studies used epidemiologically robust population based methods to assess the prevalence of trachoma [26]–[28], two of which limited analyses to acute trachoma in children [27], [28]. The fourth study used a school based sampling strategy of children aged 7–11 years [29]. The moderate quality papers all measured several climatic factors using data from weather stations (two studies) or gridded datasets (two studies). Moderate quality papers also tended to account for the possible function of time, allowing for trend and seasonal effects, and reported negative results. The lower quality papers all used robust methods to assess the prevalence of trachoma, but only assessed altitude and one did not take sufficient account of confounding [32]. All eight papers reported results for active trachoma, but only one reported a finding for chronic trachoma in adult women in Mali [26].

Two further studies that provided limited evidence of the effects of climate zone on trachoma are summarised in Table 2 [11], [33] and three further papers reported on seasonality [34]–[36].

**Table 2:** Evidence for effects of climate zone on the prevalence of trachoma.

Area, Reference.	Climate zones	Trachoma outcome	Methods	Results
Nigeria, Rabiu, 2011	River delta; rain forest; Guinean forest savannah; Sudan savannah; and Sahel.	Blindness due to trachomatous corneal scarring (CO) in adults aged ≥40 years	Cross sectional survey. Multi-stage, stratified, cluster random sampling with probability proportional to size; *sample size* 13,599	Proportion of blindness (<3/60 in the better eye attributed to trachoma): Sahel 0%**, Sudan savannah 8.3%, Guinean forest savannah 0.7%, rain forest 1.0%, delta 0%.**very small sample size. Cause-specific prevalence of blindness not reported.
Northern Territory, Australia, Tedesco, 1987	Zone 1: very dry, dusty/30–54%/11°C–29°C/<25 cm. Zone 2: mod dry, dusty/39–67%/2O°C–32°C/30–64 cm. Zone 3: Sub-tropical/45–74%/23°C–33°C/100–150 cm. Zone 4a: mod dry, dusty/30–54%/11°C–29°C/25–64 cm. Zone 4b: tropical/59–80%/23°C–33°C/80–160 cm	TF/TI (active trachoma); reported in 0–11 years and 0–21 years age groups.	Cross sectional analysis of survey data. Test for heterogeneity between zones 1–4 (Kruskal-Walhs).	TF/TI incidence highest in zones 1 and 2: 0–11 years “significantly different”, 0–21 years “statistically significant different” Zone 1: 0–11 yrs 77.9%; 0–21 yrs 57.4%; Zone 2: 0–11 yrs 56.4%; 0–21 yrs 46.7%; Zone 3: 0–11 yrs 25.0%; 0–21 yrs 19.9%; Zone 4a: 0–11 yrs 33.3%; 0–21 yrs 25.0%; Zone 4b: 0–11 yrs 18.2%; 0–21 yrs 15.4%. 0–11

### Temperature effects

Temperature was assessed in all four studies of moderate quality, giving different results. In one of the studies from Mali, the prevalence of active trachoma was significantly lower in areas with higher annual average temperature and higher sunshine fraction [28]. Monthly average temperatures however, gave different results, with hotter areas having a higher prevalence, but this did not reach statistical significance. However, in this study the range of monthly average temperatures was relatively low (tertiles: <34.6, 34.6–38.7, >38.7°C). In the study from Burkina Faso, multivariate analysis also showed the prevalence of active trachoma to be lower in areas with higher minimum temperature, with a 43% lower risk with every 1°C higher minimum temperature [29]. The other paper from Mali however, gave different results in multivariate analysis, reporting a significantly higher prevalence of active trachoma with higher mean daily temperature [26]. In Sudan, temperature did not predict the distribution of active trachoma once rainfall was included in the explanatory model [27].

### Altitude as a proxy for temperature

Altitude was used as a proxy for temperature in all eight studies (Table 1). Three studies, all of lower quality, reported a lower prevalence of trachoma at higher altitude [17], [30], [32], but two did not take account of other climatic or environmental factors [30], [32]. A further study reported the converse, with a higher odds of trachoma at higher altitude which persisted after adjusting for some confounders [31]. In the four other studies, all of which were of moderate quality and which included a range of other climate and/or environmental risk factors, altitude was dropped in final statistical models [26]–[29]. In one of these papers all study sites were at low altitude (27–669 m) [26]. In all the papers, there is likely to have been residual confounding given that altitude is a very broad indicator of temperature. This means that there is low confidence in the results, especially as the effects of altitude were not consistent.

### Rainfall

Rainfall was investigated in all four studies of moderate quality but rainfall exposures were parameterised differently. Two of the four studies gave significant findings. In the study in Sudan, long-term average rainfall was the strongest climatic predictor of active trachoma, with every 100 mm increase in rainfall being associated with a 79% lower prevalence [27]. In one of the studies in Mali, rainfall was measured as the number of rainy days, and this study also showed rainfall to lower the risk of active trachoma in children (odds ratio 0.63, 95% CI 0.41–0.97) [28]. The two other studies had weaker evidence of an association between rainfall and trachoma, as rainfall was not retained in their final statistical models as the meteorological variables were highly correlated. In univariate analyses one study reported lower odds of active trachoma with greater annual rainfall [26] while the other reported greater odds of active trachoma in children [29].

### Other variables

Relative humidity and sunshine fraction were considered by some authors but with no clear hypotheses. In one study, relative humidity was dropped from the final multivariate model as it was highly correlated with temperature and rainfall. One study found an association between sunshine fraction and active trachoma, with a lower risk of trachoma in areas with higher sunshine fractions [28]. Latitude was measured in only two studies: one of lower quality from Tanzania which did not report findings [17], and one of moderate quality from Mali. In the latter, a multivariate analysis, the latitudes 10–15°N (with higher average temperatures) were associated with a higher risk of chronic trachoma but a lower risk of active trachoma when compared with the latitude of 15–21°N as baseline [26].

### Association between trachoma prevalence and climate type

One study (mapping study in southern Sudan) that met our review criteria investigated associations between trachoma and climate type [27]. Two further studies from Nigeria and Australia [11], [33] (Table 2) were also identified: the Nigerian study reported the proportion of blindness due to trachoma in a national survey of blindness and visual impairment. Overall, the prevalence of trachoma appears to be higher in semi-arid Savannah areas where the climate is characterised by a winter dry season, a relatively short but heavy rainy summer season, and high year-round temperatures. This finding is consistent with anecdotal evidence but the association was only formally tested in the Nigerian study [33]. The Sudanese study also suggested that savannah and grassland had a higher prevalence of active trachoma than wooded savannah [27]. The prevalence of active trachoma was also reported to higher in the drier and dustier areas (zones 1 and 2) in Australia (Table 2).

### Seasonality

There is very limited evidence that active trachoma is seasonal as none of the eight studies investigated the intra-annual distribution of trachoma. Three further papers were identified which described monthly rates of active trachoma: one from Australia [34] and two from India [35], [36]. In north-western Australia, higher rates of trachoma were observed during the wet season months (14–59% in dry season compared with 46–69% in wet season) in two Aboriginal communities [34]. The Indian papers (based on surveys undertaken in 1956–63) reported monthly cases of active and chronic trachoma which showed no seasonal pattern. However, two seasonal peaks in conjunctivitis were observed in the population (in the pre- and post-monsoon periods), which were associated with observed seasonal fly abundance [37].

## Discussion

Studies designed to explore associations between climate and disease should ideally use data on temperature and precipitation that is valid for the population under study, for example, from weather station observations. For large populations and areas, gridded data from weather stations or satellite data might have to be used [38]. The latter are particularly relevant in sub-Saharan Africa where coverage by weather stations is low, although care should be taken in highland areas, where use of wrong climate data can give spurious results [39]. Altitude is often used as a crude proxy for temperature as temperatures decrease with increasing altitude. However, altitude also influences a number of other factors, including rainfall; highland areas may be wetter or drier than surrounding low land areas depending on the local climate and topography. Furthermore, vegetation, land use, and population characteristics can also vary by altitude. It is therefore important that studies assessing the influence of altitude on disease take account of these potential confounders. Temperature is also a more robust indicator than rainfall over a geographic area, due to the higher spatial and temporal variability in precipitation. Meteorological variables are often correlated in space and time and therefore it is important that the most appropriate parameterizations (including meteorological indices) are decided a priori.

This systematic literature review found evidence that low rainfall is associated with a higher prevalence of active trachoma, which is consistent with the finding that trachoma prevalence is greater in savannah areas. In one study, however, rainfall was removed from the final multivariate model due to collinearity with latitude and humidity. Low rainfall warrants further investigation as a risk factor for the distribution and prevalence of trachoma.

Despite a wealth of anecdotal information, there is very little high quality observational evidence on the role of temperature on the distribution or prevalence of active trachoma. There is even less evidence regarding chronic trachoma (and related blindness). One study reported a significant effect of lower latitude on trachomatous scarring and trichiasis in adult women in Mali [26]. Using altitude as a proxy for temperature, there is some evidence that in highly endemic areas of East Africa the prevalence of trachoma is lower at high altitudes but two papers, both risk mapping papers which also measured several co-linear variables (e.g., rainfall, humidity) did not find statistically significant associations. Although it is plausible that low temperatures have a limiting effect on the distribution of trachoma, the association could be due to confounding by social or other environmental factors. For example, in Kenya it has been observed that households of higher socio-economic status and less over-crowding tend to reside at higher altitudes than poorer households [17].

The activity and distribution of eye seeking flies may be a mechanism by which low temperatures limit trachoma prevalence, as climatic factors directly affect the seasonal activity and distribution of Muscid flies (e.g., *M. sorbens*). Laboratory studies have also shown that the lifespan of *M. sorbens* ranges from less than 12 days at 32°C to 35 days at 24°C [29], [40]. Field studies show that the distribution of *M. sorbens* is strongly correlated with altitude [41]. Climate may also effect fly activity indirectly, for example, through access to faeces for breeding, as high temperature and sunshine may induce rapid drying of faecal matter, rendering it a less effective breeding site [29].

Historically, trachoma studies have focused on changes in distribution (e.g., amongst migrant populations) and few doubted the association between dust, heat, and trachoma [42]–[44]. The systematic review found no studies that quantified an association between dust and trachoma, probably because it is difficult to measure dust exposures, especially across large spatial scales.

Only one paper reported a seasonal pattern in active trachoma [34]. Assessing seasonality in relation to trachoma is problematic for several reasons. Not only is there poor correlation between PCR-confirmed *Chlamydial* infection and the clinical signs of active trachoma, but the interval between infection and the development and resolution of clinical signs is not yet known. It is therefore important to evaluate the method of assessment of active trachoma when interpreting temporal patterns of the disease.

This review has several limitations. The quality of the climate exposure assessment in many studies was poor due to the use of broad categories (e.g. altitude) or lack of local measures for temperature and rainfall, due to limited coverage by weather stations. However, the Sudanese study used satellite data to obtain comprehensive exposures of average rainfall and temperature at a reasonable resolution, although the data would have been interpolated leading to some parameter uncertainty [27]. All the studies were undertaken in Africa, which limits generalisability of the findings. It is possible that climate-trachoma associations are location specific. For example, the relationship between trachoma and altitude may be different in Asia where overcrowding during the winter among populations living at high altitude is thought to promote transmission (B. Qureshi, personal communication). Other limitations are that some studies used relatively small sample sizes and prevalence estimates of trachoma had wide 95% confidence intervals, which reduce the power to detect statistically significant differences in outcomes. Most trachoma research is conducted either within foci where trachoma is endemic, or is undertaken to identify trachoma endemic areas where control programmes are required. This is a major limitation when exploring climatic factors where the edges of the distribution are of interest. It is also possible that some publication bias has occurred, with studies not finding an association not being published.

The current understanding of trachoma transmission is that multiple factors combine to propagate this preventable disease. Many of these factors are difficult to quantify, but are very important when considering the entire transmission cycle and interactions between socio-economic and environmental factors. The findings of this study suggest that climatic factors may also play a role in the distribution and prevalence of trachoma. Socioeconomic factors (e.g., poverty) and certain behaviours (e.g., migration) warrant further attention as they impact on those at risk of trachoma and are, in turn, affected by environmental factors [5], [45], [46]. The findings of this review support the call for greater investment in the “E” element of the SAFE strategy.

The WHO-led Alliance for the Global Elimination of Blinding Trachoma (GET2020) aims to eliminate blinding trachoma by the year 2020, and there are international partnerships, including a drug donation programme for control [4], [47], [48]. There are ongoing international efforts to map several NTDs, including trachoma, providing up to date information on the distribution of trachoma. The findings of this review are of value for those mapping the distribution of trachoma, as altitude, temperature and rainfall may be additional parameters for consideration at the planning stages. Better delineation of trachoma endemicity, which will identify the edge of endemic foci, will allow more informative studies of the impact of climate over a large scale on the distribution of trachoma.

The findings of this review add impetus to trachoma control because if the climate in sub-Saharan Africa was to become hotter and drier (due to either natural variability or anthropogenic forcing), this may potentially influence the distribution and severity of trachoma. Finally, this review highlights the relative paucity of studies exploring these potential associations and the poor quality of much of the climate data both in terms of coverage and frequency at which the data were collected.

## Supporting Information

Table S1 Search terms used to assess the impact of climatic risk factors for active and chronic trachoma.
(DOC)

Table S2 PRISMA checklist.
(DOC)
