# Supplementary material for: The Impact of Climatic Risk Factors on the Prevalence, Distribution, and Severity of Acute and Chronic Trachoma
Source: PLoS Negl Trop Dis. 2013 Nov 7;7(11):e2513. doi: 10.1371/journal.pntd.0002513 (PMC3820701; doi:10.1371/journal.pntd.0002513)
Supplement: Table S1 — Search terms used to assess the impact of climatic risk factors for active and chronic trachoma. (DOC) [file pntd.0002513.s001.doc]

**Supplementary Material. Table S1: Search terms used to assess the impact of climatic risk factors for acute and chronic trachoma.**

| **CLIMATIC FACTORS** | | |
| --- | --- | --- |
| altitude.mp. or altitude/ | ecotone*.mp. | rain/ |
| arid*.mp. | el nino.mp. | sahel.mp. |
| climat$.mp. | el nino-southern oscillation/ | savanna/ or savanna*.mp. |
| climate change.mp. or climate change/ | elevation.mp. or geographic elevation/ | season$.mp. |
| climate.mp. | enso.mp. | semiarid climate/ or desert climate/ or desert/ or ecosystem/ |
| climate/ | flood$.mp. | soi.mp. |
| cold.mp. | heat wave$.mp. | southern oscillation.mp. |
| cyclone$.mp. | heat$.mp. | temperature.mp. |
| cyclonic storms/ | heatwave$.mp. | temperature/ |
| disasters/ or floods/ | hot temperature/ or cold temperature/ | tropical climate/ or desert climate/ or cold climate/ |
| drought$.mp. | humid$.mp. | tropical storm$.mp. |
| droughts/ | hurricane$.mp. | typhoon$.mp. |
| dryland$.mp. | meteorologic$.mp. | weather.mp. |
| eco* zone*.mp. | monsoon$.mp. | weather/ or meteorological concepts/ or seasons/ or humidity/ |
| ecologic*.mp. | precipitation.mp. | wind$.mp. |
| ecology/ or ecosystem/ | rain$.mp. | wind/ |
|  | **AND** |  |
| **ACUTE TRACHOMA (TF/TI)** |  | **CHRONIC TRACHOMA (TS/TT/CO)** |
| chlamydia trachomatis/ |  | corneal opacity/ |
| chlamydia* trachomatis.mp. |  | eye* scar*.mp. |
| granular conjunctivitis.mp. | **or** | trachoma* scar*.mp. |
| trachoma$ follicular.mp. |  | trachoma* trichiasis.mp. |
| trachoma$ intense.mp. |  | trachoma*.mp. |
| trachoma$.mp. |  | trachoma/ |
| trachoma/ |  | trichiasis.mp. |
|  |  | trichiasis/ |
